# 氧化还原平衡稳态调控第三代非小细胞肺癌EGFR-TKIs耐药的研究进展

**DOI:** 10.3779/j.issn.1009-3419.2025.106.21

**Published:** 2025-07-20

**Authors:** Ting LUO, Chen FANG, Feng QIU

**Affiliations:** 330000 南昌，南昌大学第一附属医院肿瘤科; Department of Medical Oncology, The First Affiliated Hospital of Nanchang University, Nanchang 330000, China

**Keywords:** 肺肿瘤, 耐药性, 表皮生长因子受体-酪氨酸激酶抑制剂, 奥希替尼, 活性氧, 氧化还原稳态, Lung neoplasms, Drug resistance, Epidermal growth factor receptor-tyrosine kinase inhibitors, Osimertinib, Reactive oxygen species, Redox homeostasis

## Abstract

非小细胞肺癌（non-small cell lung cancer, NSCLC）是全球范围内致死率最高的恶性肿瘤之一，表皮生长因子受体-酪氨酸激酶抑制剂（epidermal growth factor receptor-tyrosine kinase inhibitors, EGFR-TKIs）的临床应用成功革新了*EGFR*突变阳性NSCLC的治疗模式，可显著延长患者无进展生存期，已成为晚期肺腺癌的一线标准治疗方案。然而，获得性耐药导致的治疗失败仍是制约临床获益的核心问题，其机制呈现高度异质性。在EGFR-TKIs耐药细胞中普遍存在“氧化应激代偿”现象，氧化还原稳态通过精确调控活性氧（reactive oxygen species, ROS）的生成与清除在肿瘤细胞增殖与凋亡的平衡中起着关键作用。本综述旨在创新性构建氧化还原平衡动态调控网络影响第三代EGFR-TKIs耐药的理论框架，着重阐释ROS在*EGFR*依赖性和非依赖性耐药机制中的多维调控作用，并深入探讨靶向ROS动力学阈值及抗氧化系统的干预策略，不仅为克服第三代EGFR-TKIs获得性耐药开辟“代谢检查点”调控的创新路径，也为构建基于氧化还原生物标志物的动态治疗决策系统奠定分子基础，推动肿瘤治疗由单一靶向抑制迈向多维度代谢重塑的精准医学新模式。

## Abstract (en)

Non-small cell lung cancer (NSCLC) ranks among the most lethal malignancies worldwide. The clinical application of epidermal growth factor receptor-tyrosine kinase inhibitors (EGFR-TKIs) have successfully revolutionized the treatment paradigm for *EGFR*-mutant NSCLC, significantly prolonging progression-free survival and establishing EGFR-TKIs as the standard first-line therapy for advanced lung adenocarcinoma. However, acquired resistance remains a major obstacle to sustained clinical benefit, with mechanisms that are highly heterogeneous. A phenomenon of “oxidative stress compensation” is commonly observed in EGFR-TKIs-resistant cells, where in redox homeostasis, through the precise regulation of reactive oxygen species (ROS) generation and elimination, plays a pivotal role in maintaining the balance between tumor cell proliferation and apoptosis. This review aims to innovatively construct a theoretical framework describing how dynamic redox regulation influences resistance to third-generation EGFR-TKIs. It focuses on the multifaceted roles of ROS in both *EGFR*-dependent and *EGFR*-independent resistance mechanisms, and further explores therapeutic strategies that target ROS kinetic thresholds and antioxidant systems. These insights not only propose an innovative “metabolic checkpoint” regulatory pathway to overcome acquired resistance to third-generation EGFR-TKIs, but also lay a molecular foundation for developing the redox biomarker-based dynamic therapeutic decision-making systems, thereby facilitating a shift in NSCLC therapy from single-target inhibition toward multi-dimensional metabolic remodeling in the context of precision medicine.

表皮生长因子受体-酪氨酸激酶抑制剂（epidermal growth factor receptor-tyrosine kinase inhibitors, EGFR-TKIs）的临床应用显著改善了*EGFR*突变阳性非小细胞肺癌（non-small cell lung cancer, NSCLC）患者的生存预后^[1]^，其通过特异性抑制*EGFR*胞内结构域酪氨酸激酶活性，有效阻断下游有丝分裂原活化蛋白激酶（mitogen-activated protein kinase, MAPK）/磷脂酰肌醇3-激酶（phosphoinositide 3-kinase, PI3K）/信号转导及转录激活因子3（signal transducer and activator of transcription 3, STAT3）等致癌信号通路的异常活化。然而，获得性耐药导致的治疗失败仍是临床面临的重大挑战，约50%接受第一/二代EGFR-TKIs治疗的患者在10-14个月内出现疾病进展，且第三代抑制剂奥希替尼的中位无进展生存期也仅为18.9个月^[2]^。耐药机制呈现时空异质性特征，既包括*EGFR* T790M/C797S二次突变等*EGFR*靶点依赖性途径，亦涉及间充质-上皮转化因子（mesenchymal-epithelial transition factor, *MET*）/人类表皮生长因子受体2（human epidermal growth factor receptor 2, *HER2*）扩增、组织学转化等非*EGFR*靶向逃逸方式。近年研究^[3]^发现，肿瘤氧化还原稳态失衡通过调控活性氧（reactive oxygen species, ROS）代谢网络，在EGFR-TKIs耐药过程中发挥关键作用。其中，核因子E2相关因子2/Kelch样ECH相关蛋白1（nuclear factor erythroid 2-related factor 2/Kelch-like ECH-associated protein 1, NRF2/KEAP1）信号轴的异常激活能够增强谷胱甘肽（glutathione, GSH）合成限速酶谷胱甘肽合成酶催化亚基（glutamate-cysteine ligase catalytic subunit, GCLC）/谷胱甘肽合成酶调节亚基（glutamate-cysteine ligase modifier subunit, GCLM）表达，促进化疗耐药相关蛋白多药耐药相关蛋白1（multidrug resistance-associated protein 1, MRP1）/乳腺癌耐药蛋白（breast cancer resistance protein, BCRP）介导的药物外排^[4]^；而调控氧化还原稳态及耐药表型形成的关键蛋白APE1/Ref-1，通过氧化还原依赖性机制激活PI3K/AKT通路，促进肿瘤干细胞特性及奥希替尼耐受表型的形成。本文将系统综述氧化还原平衡稳态调控第三代EGFR-TKIs耐药的研究进展，探讨靶向ROS动力学阈值、开发氧化还原“合成致死”策略的转化医学潜力，为克服EGFR-TKIs耐药提供创新性干预思路。

## 1 氧化还原稳态调控网络

氧化还原稳态通过精确调控ROS的生成与清除在肿瘤细胞增殖与凋亡的平衡中起着关键作用。ROS的来源主要包括内源性与外源性两类（图1）。内源性ROS主要由线粒体电子传递链（electron transport chain, ETC）和NADPH氧化酶（nicotinamide adenine dinucleotide phosphate oxidases, NOXs）驱动生成，其中ETC中的复合物I和III在电子转移过程中生成超氧阴离子自由基（superoxide anion radical, O₂•⁻）^[5]^，NOXs则通过电子转移催化生成O₂•⁻或H₂O₂。除上述两者外，内质网在蛋白质折叠过程中也可以产生ROS^[6]^，尤其在内质网应激状态下，该过程显著增强，加剧细胞氧化应激水平。X射线、紫外线以及部分化疗药物等外源性诱导因素也是ROS的重要来源，通过诱导氧化应激反应提高细胞内ROS水平^[7,8]^，导致肿瘤细胞DNA损伤。为维持细胞稳态、避免氧化损伤，肿瘤细胞依赖高度复杂的三级抗氧化防御体系对ROS进行清除与调控。超氧化物歧化酶（superoxide dismutase, SOD）家族构成第一道防线，通过催化O₂•⁻歧化反应将O₂•⁻转化为H₂O₂和O₂，实现ROS的初级清除。二级防御由过氧化氢酶（catalase, CAT）和谷胱甘肽过氧化物酶（glutathione peroxidase, GPX）家族执行，分别通过清除H₂O₂和还原脂质过氧化物维持细胞氧化还原稳态。其中，CAT作为四聚体血红素酶，在胞内pH接近7.0时活性最强，CAT表达受到miR-30a负向调控，在耐药细胞中常呈活性下降，导致H₂O₂蓄积^[9]^；GPX4通过还原脂质过氧化物，在结肠癌细胞对铁死亡诱导剂erastin的耐药形成中发挥重要作用，其过表达可以显著提高erastin的半数抑制浓度（half maximal inhibitory concentration, IC₅₀）值^[10]^，提示GPX4在erastin耐药机制中具有关键作用。三级防御包括维生素C、E和GSH等低分子量抗氧化剂，能通过清除自由基阻断链式反应，保护细胞免受持续性氧化损伤。

**图1:**
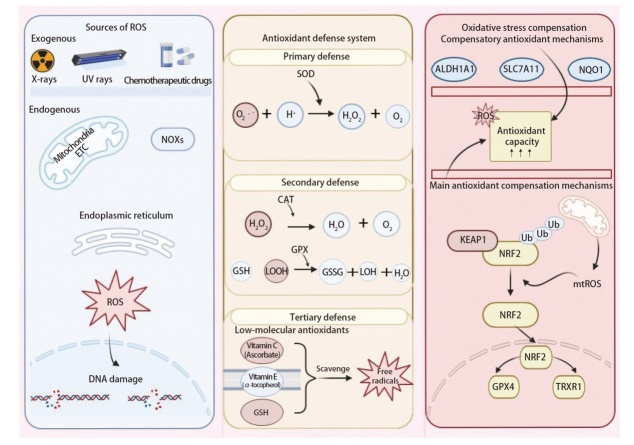
氧化还原稳态调控网络示意图。肿瘤细胞通过三级抗氧化防御体系（SOD、CAT/GPX及小分子抗氧化剂）清除内外源性ROS，以维持氧化还原稳态。耐药细胞激活以NRF2为核心的抗氧化代偿通路，上调ALDH1A1、SLC7A11与NQO1等因子，增强ROS清除能力，形成氧化应激适应性生存机制。

因此，肿瘤细胞通过建立多层次的抗氧化系统以维持ROS稳态。然而，在EGFR-TKIs治疗背景下，EGFR-TKIs耐药的肿瘤细胞中常见“氧化应激代偿”现象，即通过上调抗氧化能力抵抗ROS升高。研究^[5,11]^显示，耐药株中线粒体ROS生成显著增加，同时GPX4和TRX还原酶1（TRX reductase 1, TRXR1）的表达升高。该过程主要由NRF2/KEAP1信号通路介导，调控ROS生成和抗氧化反应，重塑有利于肿瘤生存的氧化还原微环境^[12]^。除此之外，耐药细胞还通过多种补偿性抗氧化机制增强ROS清除能力。作为一种细胞内醛类代谢酶，ALDH1A1可以通过清除脂质过氧化物及ROS诱导的活性醛类物质，降低氧化应激水平。研究^[13]^表明，在肺腺癌耐药模型中，ALDH1A1高表达能够诱导细胞对EGFR-TKIs和化疗药物产生交叉耐药。此外，SLC7A11介导的胱氨酸摄取驱动GSH合成，通过维持氧化还原稳态构成促氧化治疗的耐药屏障^[14]^；NAD(P)H:醌氧化还原酶1[NAD(P)H:quinone oxidoreductase 1, NQO1]通过消耗NADPH还原醌类毒素，阻断氧化链式反应以增强抗氧化防御。由此可见，多通路抗氧化代偿机制共同维持耐药细胞的氧化还原稳态。

## 2 EGFR-TKIs耐药机制中的ROS调控作用

EGFR-TKIs显著改善*EGFR*突变NSCLC患者的临床疗效，延长患者的生存期并减少不良反应。第三代EGFR-TKIs奥希替尼凭借其卓越的疗效和良好的安全性，已成为*EGFR*阳性突变NSCLC患者的一线治疗选择。然而，类似于第一/二代EGFR-TKIs，奥希替尼治疗后也不可避免地出现耐药现象^[15]^。获得性耐药机制分为*EGFR*依赖性（on-target）和*EGFR*非依赖性（off-target）两类（表1）。近年来的研究^[3]^发现，ROS通过调控氧化还原动态平衡，在EGFR-TKIs耐药形成发展中发挥核心作用。ROS不仅能够激活PI3K/AKT和MAPK/ERK等关键信号通路，还诱导代谢重编程及表观遗传重塑，从而维持耐药细胞的生存优势^[16]^。深入解析ROS介导的耐药网络，有助于挖掘新靶点并开发高靶向性药物，为制定精准抗肿瘤治疗策略提供理论依据。

**表1:** 基于氧化还原稳态调控的EGFR-TKIs耐药机制及其潜在治疗策略总结

Resistance mechanism subtype	ROS-driven molecular effects	Representative targets/Pathways	Potential therapeutic strategies
EGFR-dependent (on-target) resistance mechanisms			
Structural mutations	Oxidation of EGFR C797 enhances NOX2 binding; conformational changes in G796/L792 mutations may be modulated by ROS	EGFR C797, NOX2, G796, L792	Target NOX2 or modulate redox environment to prevent ROS-driven resistance-enhancing mutations
Post-translational modifications	ROS interferes with glycosylation (Asn420/579) by inhibiting α2,3-ST and FUT enzymes, prolonging EGFR membrane retention and activation	α2,3-ST, FUT, EGFR-Asn420/579, glycosylation enzymes	Glycosylation modulators or EGFR degradation enhancers to disrupt ROS-mediated post-translational activation
EGFR-independent (off-target) resistance mechanisms			
Bypass activation via receptor tyrosine kinases	ROS enhances HER2/MET phosphorylation and stabilizes RTK fusion proteins via NOX4	NOX4, HER2, MET, ALK, FGFR, NTRK	NOX4 inhibitors, dual or pan-RTK inhibitors
Bypass activation via downstream pathways	ROS activates PI3K/AKT, MAPK/ERK, and JAK/STAT through feedback NOX4 and enzyme inhibition	NOX4, PI3K, AKT, ERK, JAK/STAT	PI3K/AKT or MAPK inhibitors; redox-modulating agents to disrupt ROS amplification loop
EMT and histological transformation	ROS activates TGF-β/Snail/Twist pathways, inducing EMT and lineage plasticity (SCLC/squamous transition)	TGF-β, Snail, Twist, SOX2, TP63	ROS scavengers (e.g., NAC), TGF-β inhibitors, epigenetic modulators
Metabolic reprogramming	ROS supports mitochondrial OXPHOS and redox-survival pathways during drug-tolerant states	PGC1α, VDAC1, mitochondria, ROS	Mitochondria-targeted pro-oxidants (e.g., Elesclomol), OXPHOS inhibitors
TME remodeling-CAF-driven bypass activation	ROS promotes CAFs to secrete HGF, activating MET/PI3K/AKT pathways	CAFs, HGF, MET, PI3K, AKT	HGF/MET pathway inhibitors, CAF-targeted therapies
TME remodeling-TAM-induced immunosuppression	Intratumoral ROS gradient induces polarization of TAMs toward M2 phenotype, enhancing IL-6/TGF-β secretion	TAMs, IL-6, TGF-β, NF-κB	NF-κB or IL-6/TGF-β inhibitors, TAMs-polarization reprogramming
ROS-adaptive signaling mechanisms			
ROS signaling-NRF2/KEAP1 axis	ROS accumulation disrupts KEAP1, stabilizing NRF2, which induces antioxidant genes (e.g., HO-1, NQO1, SLC7A11), forming a redox-adaptive phenotype that supports resistance	NRF2, KEAP1, HO-1, NQO1, xCT (SLC7A11)	NRF2 inhibitors, xCT blockers (e.g., sulfasalazine), NQO1 inhibitors
ROS signaling-NF-κB and HIF-1α pathways	ROS activates NF-κB and stabilizes HIF-1α, promoting inflammatory survival signaling and hypoxia adaptation under TKIs stress	NF-κB, HIF-1α, IL-6, VEGF	NF-κB inhibitors (e.g., BAY 11-7082), HIF-1α inhibitors (e.g., PX-478), anti-IL-6 antibodies

EGFR-TKIs: epidermal growth factor receptor-tyrosine kinase inhibitors; AKT: protein kinase B; ALK: anaplastic lymphoma kinase; CAFs: cancer-associated fibroblasts; EMT: epithelial-mesenchymal transition; ERK: extracellular signal-regulated kinase; FGFR: fibroblast growth factor receptor; FUT: fucosyltransferase; HGF: hepatocyte growth factor; HIF-1α: hypoxia-inducible factor 1α; HO-1: heme oxygenase 1; IL-6: interleukin 6; JAK: Janus kinase; MAPK: mitogen-activated protein kinase; MET: mesenchymal-epithelial transition factor; RTK: receptor tyrosine kinase; NAC: N-acetylcysteine; NF-κB: nuclear factor kappa B; NOX2/NOX4: NADPH oxidase 2/4; NRF2: nuclear factor erythroid 2-related factor 2; NQO1: NAD(P)H quinone dehydrogenase 1; OXPHOS: oxidative phosphorylation; PGC1α: peroxisome proliferator-activated receptor gamma coactivator 1-alpha; PI3K: phosphoinositide 3-kinase; SOX2: SRY-box transcription factor 2; STAT3: signal transducer and activator of transcription 3; TAMs: tumor-associated macrophages; TGF-β: transforming growth factor beta; TP63: tumor protein 63; VEGF: vascular endothelial growth factor; VDAC1: voltage-dependent anion channel 1; SCLC: small cell lung cancer.

### 2.1 *EGFR*依赖性耐药中的ROS调控作用

On-target耐药机制主要取决于*EGFR*关键氨基酸残基的构象改变，此类突变阻碍奥希替尼与EGFR酪氨酸激酶结构域三磷酸腺苷（adenosine triphosphate, ATP）结合位点的结合，进而导致耐药。研究^[3]^表明，*EGFR*第20外显子第797位上的半胱氨酸残基（Cys^797^）是奥希替尼的不可逆共价结合位点，其突变是常见耐药形式；并且该残基发生氧化后还能够增强野生型*EGFR*（*EGFR*^WT^）与NOX2结合，促进ROS生成。在奥希替尼耐药NSCLC细胞系中，基础ROS和NOX2水平较高，与肺癌患者较差预后呈正相关。*EGFR* G796、L792等其他位点的氨基酸突变也被证实与奥希替尼耐药相关^[17]^。除结构突变外，ROS还通过调控*EGFR*翻译后修饰，促进*EGFR*信号持续活化。研究^[18]^表明，ROS通过氧化应激微环境间接影响糖基转移酶功能，干扰Asn420、Asn579等*EGFR*关键位点的糖基化，从而改变其跨膜区构象及胞外结构域稳定性。这种修饰障碍会促使*EGFR*异常二聚化，增强配体非依赖性激活效率。此类翻译后修饰与C797等位点突变形成“结构域突变-修饰异常”协同机制，共同维持EGFR信号通路的代偿性再激活，揭示ROS在on-target耐药机制中的多层调控地位，也为干预*EGFR*修饰相关的ROS依赖通路提供新的可能性。不过细胞内氧化应激反应对上述位点突变的生物学效应尚不完全清楚，是否增强EGFR-TKIs的耐药性仍需进一步探索。

### 2.2 *EGFR*非依赖性耐药中的氧化应激网络

相较于*EGFR*依赖性耐药机制，研究^[15]^表明*EGFR*非依赖性耐药途径在奥希替尼治疗中的发生频率更高。这可能与奥希替尼对*EGFR*靶点的强效抑制作用有关，促使肿瘤细胞通过激活其他受体酪氨酸激酶和下游信号通路等旁路途径，以及组织学转化、代谢重编程等机制形成耐药性。

#### 2.2.1 ROS与旁路激活

作为ErbB受体酪氨酸激酶家族的重要成员，*HER2*（ErbB2）与*EGFR*（ErbB1）共享PI3K/AKT/mTOR等核心下游信号轴。AURA3和FLAURA临床试验的纵向血浆分析^[19]^显示，2%-5%的奥希替尼耐药患者存在*HER2*扩增，其通过异源二聚化持续激活*EGFR*下游信号传导。值得注意的是，该扩增事件与ROS介导的*HER2*酪氨酸激酶结构域磷酸化增强存在显著相关性，提示氧化应激微环境能够动态调控*HER2*的活化状态。与之相似，*MET*扩增作为EGFR-TKIs耐药的传统旁路激活模式，在奥希替尼耐药中的发生率更高（5%-24%）。尽管*MET*扩增与EGFR-TKIs代偿耐药的关系已被广泛证实，但ROS是否通过调控*MET*基因拷贝数变异或转录后修饰参与该过程仍属未知领域。除此之外，致癌基因融合事件也是奥希替尼获得性耐药的重要驱动因素。研究^[20]^揭示，*ALK*、*FGFR3*及*NTRK1*等基因融合均可以独立诱导奥希替尼耐药表型，而ROS作为维持*ALK*激酶活性的关键调控分子，可能通过增强融合蛋白稳定性与下游信号转导效率，与基因融合事件产生协同促耐药效应。EGFR-TKIs耐药细胞常同时呈现多个受体酪氨酸激酶旁路的激活，而ROS作为多个信号通路的共同调控节点，不仅驱动各通路独立活化，还可能协调形成促耐药的信号网络。

下游信号通路的异常活化也是旁路激活的重要机制之一。EGFR通过激活PI3K/AKT、RAS/MAPK/ERK及JAK/STAT等下游信号通路，介导其促癌生物学功能^[21,22]^。ROS不仅可以直接激活上述通路，还通过正反馈上调NOX家族氧化酶表达、抑制抗氧化酶活性，形成“ROS-信号通路活化-ROS增强”的自我强化环路^[23,24]^。持续激活的AKT和ERK等效应分子上调NOX家族氧化酶的表达，同时抑制抗氧化酶的活性，达到破坏细胞内氧化还原稳态并加剧肿瘤耐药表型的目的。*RAS*突变体通过激活NOX4催化亚基，驱动线粒体膜电位去极化，引起O₂•⁻过度生成。放大的氧化应激效应不仅加速肿瘤细胞增殖，还赋予肿瘤细胞更强的耐药性。值得注意的是，针对该环路的靶向干预已有初步成效——NOX4抑制剂在体外逆转*KRAS*突变NSCLC细胞奥希替尼耐药的效果^[12]^，为ROS调控参与旁路耐药形成提供直接证据，并突出其作为潜在干预靶点的价值。

#### 2.2.2 ROS与组织学转化

肿瘤细胞可塑性作为治疗逃逸的核心机制，通过动态表型转换赋予癌细胞适应性生存优势。上皮间质转化（epithelial-mesenchymal transition, EMT）的可逆性转变过程由氧化还原信号精确调控，ROS通过介导细胞外基质重塑、细胞骨架重组和迁移能力增强等关键步骤，促进癌细胞从上皮表型转变为间质样表型。Raoof等^[25]^研究证实，在NSCLC产生EGFR-TKIs耐药过程中，吉非替尼诱导的E-钙黏蛋白下调与波形蛋白上调直接关联EMT进程。另一种细胞可塑性表现为转分化，是细胞谱系的不可逆转变，与黑色素瘤、肺癌和基底细胞癌等多种恶性肿瘤的药物耐受性密切相关。研究^[26]^报道，ROS驱动肺腺癌向鳞状细胞癌的转分化能够加速NSCLC对EGFR-TKIs的治疗抵抗，其中LKB1缺失通过抑制脂肪酸氧化与NADPH生成诱导ROS积累，进一步促进该转分化过程的发生。氧化还原信号通过调控EMT和转分化等细胞可塑性过程，在NSCLC对EGFR-TKIs耐药形成中发挥关键作用。

#### 2.2.3 ROS与代谢重编程

尽管多数癌细胞依赖无氧糖酵解（Warburg效应）以支持其快速增殖，但在耐药状态下，癌细胞的代谢特性与静息正常细胞相似，表现为更依赖于氧化磷酸化（oxidative phosphorylation, OXPHOS），或者形成既依赖OXPHOS又依赖糖酵解磷酸戊糖途径的混合代谢模式。Viale研究团队^[27]^发现，肺腺癌EGFR-TKIs耐药细胞在抑制致癌信号通路后，表现出活跃的OXPHOS与受损的糖酵解代谢特征。与药物敏感组相比，药物耐受持久组细胞的耗氧率提升约4倍。深入的转录组学和代谢组学分析发现PGC1α和VDAC1等线粒体标志物在耐药细胞中显著上调，提示线粒体代谢在肿瘤耐药机制中具有重要作用。显然，进入耐药状态的细胞会大幅激活线粒体功能，相应的代谢副产物ROS亦显著增加，以协调EGFR-TKIs耐药细胞赖以生存的氧化还原信号通路。总体而言，耐药细胞通过协调可塑性、转分化、细胞周期和代谢途径等细胞表型，以适应肿瘤治疗引发的细胞应激。这一适应性重塑过程受氧化还原信号网络调控，促使耐药细胞维持氧化还原稳态和代谢平衡。

#### 2.2.4 ROS与TME重塑

TME作为由异质性细胞与基质成分构成的动态系统，在肿瘤发生、进展及治疗抵抗中发挥关键作用。TME主要由肿瘤相关成纤维细胞（cancer-associated fibroblasts, CAFs）、肿瘤相关巨噬细胞（tumor-associated macrophages, TAMs）和内皮细胞等基质细胞组成，通过细胞间接触、可溶因子分泌与细胞外基质重构等方式诱导肿瘤细胞获得耐药表型^[28]^。最新单细胞空间转录组研究^[29]^揭示，在接受*EGFR*或*ALK*等靶向TKIs治疗的转移性NSCLC患者中，耐药阶段的肿瘤组织呈现出显著的免疫-基质重构特征，具体表现为巨噬细胞浸润比例升高、I/III型胶原蛋白沉积增加，同时伴随白细胞介素6（interleukin-6, IL-6）、转化生长因子β（transforming growth factor-β, TGF-β）等旁分泌因子的异常高表达。

进一步研究^[29]^发现，氧化应激主要通过ROS介导的基质细胞表型转化及旁分泌信号，重塑TME代谢模式，促使免疫抑制微环境形成并加速耐药性演化。CAFs作为耐药核心细胞，其分泌活性受氧化应激信号通路的调控。Lamichhane等^[30]^研究表明，在结直肠癌类器官-CAFs共培养模型中，CAFs通过分泌肝细胞生长因子（hepatocyte growth factor, *HGF*）激活肿瘤细胞MET受体，驱动PI3K/AKT信号通路活化及Cyclin E表达，促进肿瘤增殖和干细胞特性，最终导致结直肠癌对雷莫芦单抗和雷戈拉非尼等靶向药物的耐药发生。该发现与Straussman等^[31]^关于B型RAF原癌基因（v-raf murine sarcoma viral oncogene homolog B1, *BRAF*）抑制剂耐药的研究形成机制呼应，在人黑色素瘤细胞与原代成纤维细胞的共培养体系及异种移植小鼠模型中，HGF/MET轴活化同样通过PI3K/AKT信号赋予肿瘤细胞治疗抵抗能力。此外，HER2阳性乳腺癌耐药复发也与HGF/MET轴激活及巨噬细胞浸润密切相关，提示该信号通路在多种实体瘤耐药中具有普适性。

TAMs的极化状态在TME中同样发挥重要作用，M1型TAMs具有抗肿瘤功能，而M2型TAMs则促进肿瘤进展^[32]^。基于小鼠肺癌异种移植瘤组织巨噬细胞分选及表型分析的研究发现，M2型TAMs显著富集，且TAMs极化受ROS浓度梯度调控，低浓度ROS促进M2极化，而高浓度ROS则通过核因子κB（nuclear factor kappa B, NF-κB）信号通路诱导M1型极化^[33]^。TME通过细胞外基质重构与免疫细胞极化调节肿瘤应对药物选择压力的能力，而ROS作为信号中介在调控基质细胞行为与免疫应答中居于核心地位，推动耐药相关微环境的形成与维持。靶向ROS-TME相互作用网络，有望为克服EGFR-TKIs耐药提供新策略。

## 3 靶向氧化应激克服EGFR-TKIs耐药的治疗策略

ROS在肿瘤中的作用呈现双重效应：低水平的ROS促进细胞增殖、迁移及耐药性形成，在肿瘤干细胞中，ROS可以诱导P-gp等外排泵的表达，增强细胞的多药耐药性；而高水平的ROS则可以激活ASK1-JNK/p38 MAPK等通路，诱导细胞周期停滞与凋亡，从而克服EGFR-TKIs耐药。与正常细胞相比，恶性肿瘤细胞对氧化应激诱导的损伤表现出更高的敏感性，使得促氧化剂成为克服TKIs耐药的研究重点。基于此原理，促氧化治疗可以通过直接增加ROS的生成或靶向抑制癌细胞内源性抗氧化系统间接提升细胞内ROS浓度。目前，已开发应用化疗药物、小分子化合物、光疗剂等多种治疗方法，并通过调节氧化还原代谢产物或干扰关键氧化还原信号通路来增强治疗效果^[34]^。然而，要制定更加精准的治疗方案，仍需深入理解这些治疗方法的分子机制，并系统研究其应用策略。

本文总结了当前临床试验中的ROS诱导剂、抗氧化系统阻断剂的作用机制、适应证及研究现状（表2）。尽管部分ROS靶向药物在细胞及动物模型中展现出良好抗瘤活性，但多项临床试验未能取得理想效果，提示氧化还原网络存在复杂的代偿机制。从表2所列失败案例分析可见，抗氧化代偿通路的冗余性与动态激活是影响疗效的共性原因。例如，GPX4抑制剂虽然可以诱导铁死亡，但耐药细胞能够通过上调固态载体家族7成员11（solute carrier family 7 member 11, SLC7A11）缓冲ROS积累，进而削弱治疗效果；*NRF2*及其靶基因如*NQO1*、血红素氧合酶1（heme oxygenase-1, HO-1）的持续激活也进一步增强ROS解毒能力，干扰促氧化治疗效应。这些现象表明，单一靶点干预难以打破氧化还原稳态的整体屏障。未来研究应重点关注多靶点协同策略、组合用药模式以及预测性生物标志物的建立，以提升促氧化治疗的精准性与可控性。

**表2:** 采用靶向氧化应激的药物进行抗癌治疗的临床试验（数据来源：ClinicalTrials.gov，截至2025年4月15日）

Drug name	Mechanism of action	Cancer types	Study design	Recruitment status	Trial ID
ROS inducers					
STA-4783（Elesclomol）	Induction of oxidative stress and opper ionophore activity	Stage IIIB or stage IV NSCLC	Phase II	Completed	NCT00088088
		Stage IV metastatic melanoma	Phase III	Terminated	NCT00522834
Paclitaxel	Various mechanisms, including activation of NADPH oxidase and induction of ER stress	HER2-negative metastatic or locally advanced unresectable BRCA-associated breast cancer	Phase II	Completed	NCT02163694
		Chemotherapy naive epithelial ovarian cancer	Phase II	Completed	NCT00129727
		Advanced NSCLC	Phase III	Completed	NCT00540514
Cisplatin	Various mechanisms, including direct generation, mitochondrial damage, activation of NADPH oxidase, and induction of ER stress	Squamous cell carcinoma of the head and neck	Phase II	Active, not recruiting	NCT02994069
Drugs targeting GSH system					
NOV-002	An oxidized glutathione analog that modulates the intracellular GSH/GSSG ratio	Platinum resistant cancer of ovarian origin	Phase II	Completed	NCT00345540
		HER2-negative stage IIB-IIIC breast cancer ovarian cancer	Phase II	Completed	NCT00499122
		NSCLC	Phase III	Completed	NCT00347412
L-asparaginase	Various mechanisms, including hydrolyzing glutamine and GSH depletion	Locally advanced or metastatic pancreatic adenocarcinoma	Phase I	Completed	NCT04292743
		Progressive metastatic pancreatic carcinoma	Phase II	Completed	NCT02195180
		Ductal adenocarcinoma of the pancreas	Phase III	Completed	NCT03665441
Buthionine sulfoximine (BSO)	Irreversible inhibitor of GCLC that inhibits de novo GSH synthesis	Resistant or recurrent high-risk neuroblastoma Progressive neuroblastoma that has not responded to previous therapy	Phase I Phase I	Completed Completed	NCT00005835 NCT00002730
Telaglenastat hydrochloride (CB-839 HCl)	Inhibitor of glutaminase, interfering with glutamate-dependent cellular metabolism and redox status	Solid tumors Advanced hematologic malignancies Leukemia	Phase I Phase I Phase I	Completed Completed Completed	NCT02071862 NCT02071888 NCT02071927
		Advanced myelodysplastic syndrome	Phase I	Completed	NCT03047993
		Metastatic and refractory RAS wildtype colorectal cancer	Phase I/II	Completed	NCT03263429
		stage IV NSCLC	Phase I/II	Active, not recruiting	NCT03831932
		Solid tumors	Phase I/II	Completed	NCT03965845
		Melanoma, ccRCC, NSCLC	Phase I/II	Terminated	NCT02771626
		Advanced or metastatic solid tumors with specific mutations	Phase II	Active, not recruiting	NCT03872427
		Advanced or metastatic RCC	Phase II	Completed	NCT03163667, NCT03428217
Sulfasalazine	Approved as anti-inflammatory agent, competitive inhibitor of Cys/Glu transporter xCT, inducing lipid peroxidation and ferroptosis	Acute myeloid leukemias Recurrent glioblastoma Metastatic colorectal cancer	Phase I/II Phase I Phase I	Recruiting Completed Recruiting	NCT05580861 NCT04205357, NCT01577966 NCT06134388
Withaferin A	Inhibitor of GPX4	High-grade relapsed or metastatic osteosarcoma	Phase I/II	Unknown status	NCT00689195
Drugs targeting TRX system					
PX-12	Inhibitor of thioredoxin-1 (TRX-1)	Advanced carcinoma of the pancreas (stage IV disease only)	Phase II	Terminated	NCT00417287
Auranofin	Approved as antirheumatic drug, with activity targeting TrxR	Recurrent ovarian cancer Recurrent or extensive SCLC; recurrent NSCLC	Phase II Phase I/II	Active, not recruiting Completed	NCT03456700 NCT01737502
		Relapsed or refractory CLL	Phase II	Completed	NCT01419691
		Recurrent glioblastoma	Phase I/II	Completed	NCT02770378
Ethaselen	Selective inhibitor of TrxR	High-TrxR-expressing advanced NSCLC	Phase I	Completed	NCT02166242
Drug name	Mechanism of action	Cancer types	Study design	Recruitment status	Trial ID
Drugs targeting redox signaling pathways					
PX-478	Downregulate the expression of hypoxia-inducible factor 1α (HIF-1α) and its transcriptional activity	Advanced solid tumors or lymphoma	Phase I	Completed	NCT00522652
Drugs targeting NADPH generation system					
RRx-001	Novel epigenetic modulator that produces NO and ROS under hypoxic conditions and inhibits G6PD and NLRP3 activity	Advanced solid tumors or lymphomas for which there are no currently accepted curative therapies Brain metastases Newly diagnosed glioblastoma and anaplastic gliomas	Phase I Phase I Phase I	Completed Completed Completed	NCT01359982, NCT02518958 NCT02215512 NCT02871843
		Metastatic CRC	Phase II	Completed	NCT02096354
		Lung cancer, ovarian cancer and neuroendocrine tumors	Phase II	Completed	NCT02489903
		Third-line or beyond SCLC	Phase III	Terminated	NCT03699956
		SCLC	Phase III	Active, not recruiting	NCT05566041

NSCLC: non-small cell lung cancer; HER2: human epidermal growth factor receptor 2; BRCA: breast cancer susceptibility gene; ER: endoplasmic reticulum; ccRCC: clear cell renal cell carcinoma; RCC: renal cell carcinoma; CLL: chronic lymphocytic leukemia; CRC: colorectal cancer.

### 3.1 ROS诱导剂

随着医学界对氧化还原稳态认识的不断深入，基于氧化应激和氧化损伤的抗肿瘤治疗已取得显著效果。许多美国食品药品监督管理局（Food and Drug Administration, FDA）批准的抗癌药物通过增加ROS的产生来有效杀伤癌细胞，与细胞抗氧化能力增强时药物抑瘤效果的减弱相佐证。Vinca生物碱、紫杉烷类等具有干扰细胞分裂作用的微管蛋白抑制剂以及5-氟尿嘧啶、铂类等抗代谢类药物，通过破坏线粒体功能触发ROS产生^[35,36]^。近年来，一些新型小分子化合物及传统药物再利用策略已展现出优越的促氧化活性，并带来显著治疗效益。伊利司莫（STA-4783）是一种人工合成的小分子化合物，在黑色素瘤、乳腺癌等多种恶性肿瘤中表现出抗癌活性。伊利司莫能够螯合铜离子并将其转运至线粒体，从而提高细胞内ROS水平，并引起奥希替尼适应性耐药细胞的凋亡^[37]^。为评估伊利司莫单独或与传统化疗药物联合治疗多种癌症的安全性和有效性，已有多项临床试验（如NCT00522834、NCT00088088）正在进行，显示其具有巨大的实际应用潜力。

### 3.2 抗氧化系统阻断剂

除了直接型ROS诱导剂外，靶向细胞内抗氧化系统并破坏其平衡的促氧化策略也可以有效诱导氧化应激。抗氧化过程主要涉及ROS的中和与二硫键形成的逆转，这一过程主要依赖于GSH和TRX两条代谢途径的调节^[38]^。研究^[39]^表明，调节或干扰氧化还原信号的分子有望为多种疾病，特别是癌症，提供可行的治疗靶点。

#### 3.2.1 靶向GSH代谢

GSH是由甘氨酸、半胱氨酸和谷氨酸组成的三肽，是细胞内含量最丰富且必不可少的抗氧化剂。还原型GSH是其主要活性状态和存在形式。当细胞暴露于ROS时，GSH的半胱氨酸巯基可以作为电子供体，将还原型GSH转化为其氧化形式的GSH二硫化物，从而保护细胞免受氧化应激的损伤^[40]^。Gana等^[41]^研究发现，新型的小分子调节剂MRP1通过选择性增强还原型GSH的外排，导致细胞内GSH耗竭，进而诱导氧化应激、升高ROS水平并削弱癌细胞的克隆能力。此外，他们还发现ROS清除剂N-乙酰半胱氨酸能够缓解该类细胞毒性反应，提示GSH水平升高有助于癌细胞获得氧化抗性，而细胞内GSH的耗竭则有助于克服耐药性并改善癌症治疗效果。上述研究强调细胞内GSH浓度作为调控氧化还原稳态的关键节点，在决定肿瘤细胞对ROS诱导性治疗响应中的重要作用，也为发展以GSH代谢为靶点的耐药干预策略提供理论依据。

#### 3.2.2 调节TRX代谢

TRX-PRX途径是除GSH系统外最为关键的抗氧化系统之一，通过清除ROS、调节氧化还原酶活性，并与GSH氧化还原系统协同作用，维持细胞内氧化还原稳态。该系统由TRX、TRXR和NADPH组成。当TRX系统受到抑制时，细胞内会发生氧化应激。TRX系统的抑制剂能够通过增加氧化应激来诱导癌细胞死亡，从而抑制肿瘤的发展和进展^[42]^。金属配合物、硒化合物、青霉素衍生物及黄酮类化合物等多种天然或合成的TRX抑制剂已开发用于抗癌治疗。然而，TRX抑制剂的临床试验尚处于起步阶段，其疗效和安全性仍需进一步评估。值得一提的是，TRX抑制剂PX-12在体外和体内实验中均表现出显著的抗肿瘤活性。研究^[43,44]^表明，PX-12通过对TRX的Cys73残基进行不可逆的硫烷基化修饰，抑制其氧化还原活性。虽然PX-12在临床前研究和I期试验中表现出良好效果，但在II期试验中未能达到预期的抗肿瘤效果，仍需进一步研究来探索TRX系统在EGFR-TKIs治疗恶性肿瘤过程中发挥的生物学作用及其潜在分子机制。

#### 3.2.3 调节NADPH代谢

GSH和TRX对高ROS水平的调节依赖于NADPH的产生。在H_2_O_2_还原过程中，GSH转化为其氧化形式GSSG，GSSG经过NADPH依赖的谷胱甘肽还原酶还原回GSH。此外，GSH还作为底物参与TRX-S2、TRXR1和TRXR2的产生。NADPH还能够与CAT结合，维持其抗氧化活性^[45-47]^。因此，通过下调NADPH水平来降低癌细胞清除ROS的能力，有望减弱其ROS缓冲能力，引起细胞死亡。作为细胞质中催化NADP生成的关键酶，NADK通过其与*NUAK1*的直接相互作用并磷酸化其丝氨酸64（S64）位点，缓解奥希替尼诱导的ROS积累，进而促进NSCLC细胞耐药形成。而NADK-S64磷酸化抑制剂T21195与奥希替尼产生协同作用，通过诱导ROS积累逆转NSCLC的获得性耐药^[48]^。

#### 3.2.4 干扰氧化还原信号转导

氧化还原稳态的维持依赖于多个ROS敏感性信号通路的协同调控，其中以NRF2-KEAP1、NF-κB及缺氧诱导因子-1α（hypoxia-inducible factor-1 alpha, HIF-1α）通路为代表^[49]^，在肿瘤细胞应对氧化应激、调节代谢重编程及维持生存优势中发挥关键作用。NRF2作为抗氧化应答的核心转录因子，其激活可以通过解离KEAP1介导的泛素化降解，从而诱导*NQO1*和*HO-1*等下游靶基因的表达^[50]^，使肿瘤细胞更能耐受氧化应激并抵抗ROS引发的细胞死亡。然而，目前临床尚缺乏特异性靶向NRF2/KEAP1通路的小分子抑制剂。ROS也可以通过激活IκB激酶（IκB kinase, IKK）复合物，促进IκB磷酸化与降解，进而释放NF-κB入核，诱导SOD2、GPX等抗氧化与促存活基因的转录。NF-κB通路不仅传统上与炎症应答密切相关，而且在维持肿瘤细胞氧化还原稳态、调控ROS水平以及抵御EGFR-TKIs治疗压力中的功能亦日益受到关注。已有研究^[51]^表明，抑制NF-κB信号会削弱细胞的抗氧化防御能力，增强ROS的蓄积效应，从而协同TKIs治疗诱导细胞凋亡。此外，HIF-1α作为低氧诱导转录因子，在ROS环境中会被稳定化并积聚，激活血管内皮生长因子（vascular endothelial growth factor, *VEGF*）、葡萄糖转运蛋白1（glucose transporter 1, *GLUT1*）、丙酮酸脱氢酶激酶1（pyruvate dehydrogenase kinase 1, *PDK1*）等靶基因表达，促进血管生成与代谢重编程，增强细胞对缺氧及氧化胁迫的适应性。小分子HIF-1α抑制剂PX-478能够下调其转录活性并抑制*VEGF*和*GLUT1*等靶基因的表达，从而增强ROS诱导的细胞毒性^[52]^。综上，NRF2、NF-κB与HIF-1α三条ROS响应通路在EGFR-TKIs治疗耐药背景下协同构建促生存的氧化还原适应网络，通过提升抗氧化能力、抑制细胞凋亡与重塑代谢环境，共同维持肿瘤细胞的耐药稳态。靶向干扰该类信号轴，有望诱导致死性氧化应激，为克服耐药提供新的治疗策略。

### 3.3 精准靶向与个体化治疗

促氧化治疗的核心任务是将抗癌药物精确递送到靶区，避免对正常组织的损伤。纳米药物通过增强渗透性和滞留效应，实现对肿瘤组织的精准递送，从而助力个体化治疗。特异性修饰纳米颗粒可以提高药物的生物相容性、稳定性和循环时间，增强治疗效果。三苯基膦与多柔比星的结合等线粒体靶向药物成功通过线粒体途径诱导ROS生成和细胞凋亡。ROS的动态检测与调控成为癌症治疗的重要工具，通过MitoSOX和GSH/GSSG比值等探针评估肿瘤细胞的氧化还原状态，为精准治疗策略提供依据。个体化分子靶向治疗通过明确癌症驱动基因的生物标志物实现精确药物输送^[53]^。靶向金纳米颗粒递送系统结合低温光热治疗和声动力治疗，可以在肿瘤区域生成大量ROS，显著增强治疗效果^[54]^。超分子纳米平台（HA-BPY-GEF-NPs）响应近红外光和酶双重刺激，通过ROS生成和酶切割机制释放活性药物，在EGFR-TKIs耐药模型中恢复耐药细胞对TKIs的敏感性，显著增强治疗反应^[55]^。此平台有效提高治疗靶向性，减少对正常组织的毒性负担，推动个体化治疗向精准高效方向发展。结合ROS动态检测与精准输送技术，促氧化治疗在提升疗效方面展现出显著优势，尤其在克服耐药、实现药物精准递送及增强治疗安全性方面具有广阔的临床应用前景。

## 4 总结与展望

本综述系统梳理氧化还原稳态在EGFR-TKIs耐药形成中的关键作用，深入阐释ROS生成与清除之间的动态平衡机制及其在细胞命运调控中的生物学意义。相较以往聚焦单一耐药机制或ROS功能的综述，本文以*EGFR*依赖性与非依赖性两类耐药路径为主线，系统归纳出ROS在旁路激活、组织学转化、代谢重编程及TME免疫重塑中的核心功能，并提出以“氧化还原稳态”为整合视角构建多维干预框架。该框架涵盖ROS诱导剂、抗氧化系统抑制剂及干扰ROS相关信号通路的小分子药物，旨在实现针对耐药异质性的精准促氧化治疗。在此框架下，ROS不再被视为单一毒性因子，而被重新定义为维系耐药稳态的核心枢纽，并提出了基于ROS干预策略的多维联合治疗思路，体现出本文在干预靶点整合性与理论体系建构方面的创新价值。尽管ROS靶向治疗在基础研究中取得一定进展，其临床转化仍面临多重挑战。首先，ROS水平的调控具有显著的时空依赖性与细胞特异性，目前尚缺乏针对不同肿瘤亚型与治疗阶段的统一ROS阈值标准，显著限制促氧化治疗的广泛适用性。其次，NRF2/KEAP1、HIF-1α、NF-κB等氧化还原信号通路交互复杂，机制识别与干预节点尚不明确。此外，现有ROS靶向药物存在毒性大、选择性差、精准性不足等问题，且缺乏能够用于监测和患者筛选的特异性标志物。未来研究应聚焦以下方向：（1）整合多组学数据构建ROS代谢图谱，识别耐药关键节点；（2）结合人工智能（artificial intelligence, AI）与系统生物学构建调控网络，推动机制研究向靶点发现与药物设计转化；（3）开发可视化探针与响应型递送系统，实现ROS的动态监测与精确干预。临床层面，需建立融合*EGFR*状态、耐药机制与代谢特征的分层干预体系，推进诊疗一体化。总体而言，氧化还原稳态作为EGFR-TKIs耐药的核心调控机制，正在成为NSCLC治疗的重要突破口。深入解析ROS与耐药之间的因果关联，构建机制明确、策略可行的干预体系，有望为延长患者生存提供理论支持与实践方向。
